# On Alan Armstrong's ‘Towards a Strong Virtue Ethics for Nursing Practice’

**DOI:** 10.1111/nup.70027

**Published:** 2025-04-24

**Authors:** Roger Newham

**Affiliations:** ^1^ Department of Nursing and Midwifery School of Medicine and Health Sciences, College of Medicine and Health, Medical School Building University of Birmingham Edgbaston UK

**Keywords:** ethics, morality, nursing ethics, virtue, virtue ethics

## Abstract

Armstrong's (2006) ‘Towards a strong virtue ethics for nursing practice’ is focused on how the practice of nursing necessitates morally good character traits as virtues including the intellectual virtue *phronesis*. Because of this, he claims, nursing ethics should also be grounded in virtue ethics. Illness creates a unique phenomenon that involves a special therapeutic as helping relationship necessitating good interpersonal skills and patient‐centred care that, for the role of a nurse and nursing ethics, requires a focus on persons and relationships, character and emotions. Obligation, act centred normative theories are, according to Armstrong, incomplete and inadequate for nursing practice. They are incomplete and inadequate as moral theories because they ignore, or at least do not give appropriate moral importance to, other factors of life such as character, moral education, emotions and relationships. Armstrong grounds his virtue ethics in a ‘moralised’ *eudaimonia*. This leads to problems of getting from good for to good. It is suggested a non *eudaimonistic*, virtue ethics by Swanton might be just what Armstrong is after but as an account of ethics not morality.

Armstrong (2006) ‘Towards a strong virtue ethics for nursing practice’ is focused on how the practice of nursing necessitates morally good character traits as virtues including the intellectual virtue *phronesis*. Because of this, he claims, nursing ethics should also be grounded in virtue ethics. Virtue theory is an account of what virtues are, and virtue ethics grounds ethics on virtue evaluation (Driver 1999). It is a summary of the structure of his 2007 book and where necessary, referral to the book is used to expand on claims in the paper.

The 2006 paper gives an overview of why nursing practice needs the virtues and why only a virtue ethics will do. In outline it is claimed, like Pellegrino and Thomasma (1981, 1994) amongst others, that illness creates a unique phenomenon that involves a special therapeutic as helping relationship necessitating good interpersonal skills and patient‐centred care that, for the role of a nurse and nursing ethics, requires a focus on persons and relationships, character and emotions. Obligation, act centred normative theories are, according to Armstrong, incomplete and inadequate for nursing practice. They are incomplete and inadequate as moral theories because they ignore, or at least do not give appropriate moral importance to, other factors of life such as character, moral education, emotions and relationships. He claims, citing Anscombe (1958) and MacIntyre (1985), that relationships, in this case based on dependence of a patient upon a nurse, have been ignored in both moral philosophy and in nursing ethics. Additionally, obligation act‐centred moral theories fail to provide clear action guidance, over focusing on the role of moral obligations in morality which assumes decision making will always have a right answer in part because all moral actions must be at least permissible, and thus neglect or disbelieve such things as dilemmas, especially irresolvable and tragic dilemmas as well as ignore the everyday experience of moral remainder (Armstrong 2007, 75). Moral remainder is roughly the idea that even when a right moral decision and action have been made and carried out, there is still room for at least some emotional response, if not regret, for the road not taken. The result for nursing is that

…the reality of clinical nursing is not acknowledged by obligation‐based moral theories…Nurses are left without the necessary conceptual tools with which to clarify and resolve the numerous complex, multidimensional moral conflicts.

(Armstrong 2006, 117)

Yet, such theories are still taught to nursing students and used in nursing ethics.

Terms frequently seen in the nursing literature as important to nursing such as good character, emotions, partiality, relationships and holistic person‐centred care do all at least resonate with virtue ethics. Whether such resonance, and to what extent it, becomes a part of or appropriately recognised by normative moral theory is contested. But there is much that is plausible in Armstrong (2006, 2007) about nursing practice, nursing ethics and the connection to virtues, such as the emphasis on character and action, as well as emotions, and their importance for moral philosophy.

For Armstrong the emphasis is, as his title suggests, a strong one towards the necessity of the virtues, *replacing* deontic concepts of right and wrong as well as morally obligatory with the aretaic concept of acting well or excellently. He rules out hybrid, or supplementary accounts that include virtues as well as principles and rules as being theoretically unstable. However, to say an act is morally right indicates it is morally obligatory or permissible (Hacker‐Wright 2010), thus replacing deontic terms would mean replacing the moral ought and morally permissible as well as right (and impermissible and wrong). As mentioned by certain people working in virtue ethics (even if some may not use the label) such as Hursthouse, McDowell and Anscombe, the idea of doing ethics without or at least without such a strong focus on morally right, morally obliged and morally permissible is somewhat on the back foot within philosophy (or at least used to be). It is to Armstrong's credit that he makes such an attempt within nursing ethics because literature about virtue ethics still remains small and underdeveloped, especially when compared with education and business (Ferkany and Newham 2019).

However, there seems to be a problem with Armstrong's replacement of deontic concepts with aretaic if he also holds to a virtue ethics criterion of right action. For virtue ethics, the rightness of an act depends fundamentally on the virtues. Right action is virtuous action (Smith 2018). An action is right because it is virtuous, because of its v‐properties (Smith 2018) and the virtue is not to be analysed in terms of rightness. In Armstrong (2006), he states that Hursthouse does provide a criterion of right action (even if, as Hursthouse claims, under pressure to be recognised as a third moral theory) and one that includes rules as ‘virtue‐rules’, but it is unclear if it means he also agrees that there is such a criterion. If he does include it, then it is problematic in that he also claims to be replacing deontic concepts.

Virtue ethics may be understood as either without the criterion of right action or with it (Hacker‐Wright 2010). And as Hacker‐Wright (2010, 211) points out, it is the need to state at least the permissibility of actions that drives the pursuit of virtue ethics criterion of moral rightness. ‘…it would be of no interest to anyone to provide a criterion of moral rightness without those deontic implications’. So maybe it is a slip on Armstrong's part interpreting Anscombe (1958) to feel the need to replace deontic concepts rather than show how agent evaluation is strongly connected with act evaluation (Hacker‐Wright 2010). Much more strongly connected than in current obligation act‐centred moral theories. And see Crisp (2004) as the claim that Anscombe may have been wrong about ancient philosophers such as Aristotle, not having a sense of ought as being bound and hence nothing like the moral ought of modern moral philosophy. Prominent virtue ethicists such as Slote, Hursthouse and Swanton, whom Armstrong categorises as strong virtue ethicists, do attempt to provide a criterion of right action albeit in terms of virtue. But if Armstrong does not hold to a virtue ethical account of right action, but replaces the deontic with the aretaic, then his virtue ethics is quite radical (Brewer 2009) in its ethical not moral outlook (Swanton 2016, 2021). And, I think, it will have particular problematic implications for his claims about moral decision making as well as agent evaluation in nursing practice and nursing ethics. But they may only seem problematic because of the hold on moral philosophy by the deontic.

However, Armstrong's justification of virtue ethics may be based on criticisms that virtue ethics is incomplete and inadequate because it has no criterion of right action. It is thought to be problematic for at least three reasons (Hacker‐Wright 2010, 210).

First, without a criterion of moral rightness, virtue ethics would be limited to agent evaluation and could not give an adequate account of act‐evaluation. Second, it would not be action guiding. Third, it would not be able to offer an account of the intuitively plausible distinction between morally good and morally right action.

(Hacker‐Wright 2010, 210)

Armstrong (2006, 2007), claims it is obligation act‐centred moral theories that are incomplete and thus inadequate, and he counters all three difficulties above showing how virtue ethics can be a third moral theory that is especially suitable to the reality of nursing practice and thus nursing ethics. Although the idea of it being a third moral theory may again be a mistake based on the felt need to compare virtue ethics with post‐enlightenment moral theories.

This paper will assume for the sake of argument Armstrong is correct in his criticism of obligation, act‐centred normative theories and examine his strong virtue ethics and its application to nursing practice and nursing ethics. This will require a particular emphasis understanding morality as narrower than that of ethics (even though Armstrong equates the two) and its implications for the role of a nurse and action guidance for nurses.

The first general thing to mention is that Armstrong (2006) crams a lot of difficult yet essential points into the short (2006) paper making it necessary (for me at least) to read his book (2007) which in turn, I think, is very useful as a way into the large philosophical literature on virtue ethics to really gain an understanding of the distinctiveness of virtue ethics in addressing the old question of ‘How should one live?’ from where we are today. Armstrong is, in his paper and book, addressing a range of interrelated theoretical and practical issues such as how ethics itself is to be understood, especially how it relates to morality, and what can be expected from it in actual, everyday practice, especially nursing practice and nursing ethics.

Though Armstrong does not give an explicit virtue ethical account of right action, he does say what *makes* a character trait a virtue rather than a vice and this is the idea of *eudaimonia*. His focus from the beginning is on the reason for the practice of nursing and the role of the nurse and how it is suited to the idea of not just virtue but virtue ethics. The claim begins with ideas of illness creating vulnerability and dependence on others, including nurses, for help ‘…to survive, recover and fare well during and beyond illness’ (Armstrong 2006, 112). Armstrong equates faring well with *eudaimonia*.

The claim, which Armstrong (2006) makes, that helping patients fare well beyond illness is a part of a nurse's role is hard to understand, and it can be questioned if is empirically or even ideally correct. Of course, many nurses are in the business of health promotion and prevention of illness and thus deal with people ‘beyond illness’ if this means those of us who are not ‘ill’. Apart from this, I am not sure what beyond illness can mean except perhaps after death. But faring well without any restrictions to health seems close to the idea and ideal of *eudaimonia* understood as ‘flourishing’ or ‘happiness’ of human beings over a whole life and possibly after death. *Eudaimonia* is ‘a property of lives’ for the one living it (LeBar 2017). Such a concept or ideal cannot be the remit of nurses but the remit of all (Holland 2010). Its focus or focal point is the *eudaimonia* of oneself. Armstrong amongst others including Hurstbourne (1999) has to stretch the term *eudaimonia* as used by Aristotle so that the neo in neo‐Aristotelian virtue ethics approach is very much to the fore. Modern philosophers do not hold to the strict accounts of who were the virtuous and that the claim that the majority of people were not and could not be (Taylor 1988). This point should be borne in mind when criticisms of modern moral philosophy or/and the Enlightenment project are raised within nursing ethics.

Armstrong (2006, 112) is clear that the role of a nurse requires (at least sometimes) a therapeutic nurse–patient relationship with a focus on patient‐centred, collaboration to help the patients survive and recover from illness, promote the patient's independence and in terminal illness alleviate symptoms such a pain and promote dignity. Note now the restriction on faring well to what for the most part seem to be things *good for* the patient, their welfare restricted to aspects of (ill)*health*. Good for as faring well, including health as a part of this, is or at least can be distinct from morally good. At least it perhaps can be when viewed through the eyes of modern moral philosophers and also nurses. Though it is very plausible that practical reason requires that we act on/from reasons and that reasons must recognise value (good) (Raz 2001) Armstrong stipulates that faring well (*eudaimonia*) is a moralised concept. This is to counter the idea that ‘bad’ or ‘vicious’ people can fare well or even that one could be just technically competent and be a good or excellent nurse. But some connection must be made between what is good for a human being and what is *morally* good. We need to know how Armstrong is understanding moral in the moralised notion of faring well.

Normatively *eudaimonia* is reason giving and is what makes a character trait a virtue and virtue sets the substantive nature of *eudaimonia*. The virtues are reciprocally related to practical wisdom (*phronesis*) such that one cannot have virtue without *phronesis* and *phronesis* helps determine the mean which just is a character trait as virtue (LeBar 2017). *Phronesis* also has as its focus *eudaimonia* a grasp of what the good life is and there is no grasping this without the virtues (Le Bar 2017). So *eudaimonia* is normative but this does not get us all the way from practical reason to a moralised notion of faring well (*eudaimonia*).

Armstrong follows Hurstbourne's (1999) neo‐Aristotelian account of human function and thus there is a problem of how our rationality is related to us as a species and the four natural ends for human beings. Getting from good for to good is not clear at least in a moralised sense where moral is taken in a narrow scope criticised by, for example, Williams (1985) and nor is it clear in Hurstbourne (1999) where moral is perhaps not so understood. But the point for now is how this fits with a nurse's role and nursing practice.

It is plausible that nurse's role has a focal point in health. This is seen by Aristotle as an external good but necessary though not sufficient for *eudaimonia*. That helping people (for us today) is good (generally) and more specifically relieving suffering. But nurses like most healthcare professionals these days cannot achieve this on their own. Additionally, their helping and helping to relieve suffering may not reach the mark for virtuous activity. A nurse is not being generous, or especially kind in helping people he is paid and employed to help. But more tellingly for a strong *eudiamonistic* virtue ethics is that virtues are about living one's life. Happiness/flourishing is in doing this and one cannot do this for other people, though one can of course help them.

A moralised account of faring well is hard to justify and particularly so in nursing practice as a distinct and *secular* nursing ethics. Authors Armstrong cites in support such as Anscombe, the later MacIntyre and Pellegrino claim the need for an ultimate ground in Religion, and specifically Catholicism to support their moralised notion of faring well as human beings. And, as Armstrong notes, this can cause problems in nursing such as the doctrine of double effect (see also Kopelman (2019) for such a problem in the work of Pellegrino).

Armstrong uses the term acting well rather than acting rightly. It is important to note at this point the radical nature of this. Armstrong is not replacing rightness with acting well because there never was a coherent account of moral rightness to begin with. Especially one that meant actions could also always be given a right verdictive judgement such as obliged, permitted or forbidden. As stated above, whether Armstrong, following Anscombe (1958), is correct on this see, for example, Crisp (2004, 2015). Acting well and faring well, may be able to do without the deontic but is it true as Armstrong suggests, we in nursing use such terms all the time? And when we do are we clear as to what it implies. For example, faring well as *eudaimonia* or faring well mentally or physically here and now with the focus narrowed to health? There is another account of virtue ethics that does not rely on *eudaimonia* for its ground and understands *phronesis* as ethics rather than morality that emphasises practical reasoning which, I think, could support Armstrong's claims about nursing ethics. This is Swanton's (2016, 2021) pluralistic, target‐centred, virtue ethics more on which later.

Armstrong (2006, 2007) is on much stronger ground when he (following Hursthouse and MacIntyre) addresses the worries mentioned in Hacker‐Wright (2010), repeated below, that virtue ethics needs a criterion of right action or else:

First, without a criterion of moral rightness, virtue ethics would be limited to agent evaluation and could not give an adequate account of act‐evaluation. Second, it would not be action guiding. Third, it would not be able to offer an account of the intuitively plausible distinction between morally good and morally right action.

(Hacker‐Wright 2010, 210)

Despite the idea of there being no coherent account of right action Armstrong is at pains to show how a strong virtue ethics can be action assessing *and* action guiding in fact more so than obligation act‐centred theories. By connecting what it is to be a virtue (or vice), he can show how character traits are both agent assessing and action assessing. For example, unjust tells us something about the agent and about the act. Such ‘thick’ ethical concepts have a descriptive and evaluative point to them that are entangled of necessity. Armstrong seems to make such virtues as ‘just’ or ‘kind’ instrumental to a nurses and patients faring well (*eudaimonia*) though this cannot be enough; obligation act‐centred moral theories can and do this. They are more plausibly constitutive, at least at the theoretical level. But whether constitutive or instrumental virtue ethics can have something to say about agents and actions.

In regard to action guidance, Armstrong does not dispense with rules, using virtue‐rules (Hurstbourne 1999) he shows how they can help to guide action, much the same as deontological rules but from a different ground. However, such rules are not codifiable and cannot provide an algorithmic decision procedure. It is hard to gauge just how much codifiability Armstrong rejects when he claims his account is contextualist, particularist and relational. But it does run a risk of relativism (more on this below) and unjustifiable partiality.

But it is not just that v‐rules are problematic for action guidance, with virtues there is no clear guidance, because an action may encompass two or more virtues and each of the virtues may be held to a lesser or greater degree though never becoming a vice. Armstrong is explicit that virtue ethics can provide greater action guidance than obligation act‐centred theories. He cites Anscombe's (1958) use of unjust as an example which having a descriptive component as well as evaluative seems to give more guidance. But Anscombe made the point that one could not say a man was unjust and the action was nevertheless right, criticising modern moral philosophy for apparently being able to do just that. Anscombe held to moral absolutes as well as Religion and the idea that virtues could not conflict so making it conceptually impossible for an unjust man to be bad man or an unjust act to be also right (Crisp 2004). I do not think Armstrong does hold to moral absolutes nor (in any strong sense) the unity of the virtues. And so, it remains puzzling to me how he thinks virtue ethics provides better action guidance by being ‘thick’ concepts. It seems rather that the nature of the virtues makes things very complicated for action guidance and assessment where there can be constant disagreement between, for example, nurses and others. Perhaps Armstrong is changing the very idea about what is expected from action guidance when he states nurses have to ‘think hard’ about ethical concerns in nursing practice. And perhaps the decision lies in perception of the virtuous person. But of course, in healthcare, like life more generally, there may be many virtuous people involved with different perceptions.

The emphasis on just one virtue in Armstrong (2006) is not a good example because, as he says, it oversimplifies the truth. In fact, it oversimplifies it so much that it hides a potential problem for virtue ethics and its notion of overall virtuousness, and thus for action guidance, that of the enumeration problem (Russell 2011). Virtues are understood in the plural and there do seem to be many, perhaps indefinitely many of them. Armstrong (2007) does not think it helpful to say how many there are, thus it is unclear how virtue ethics can give an account of right action or if right action is being dropped how it can account for what is overall virtuous or what it is to be a virtuous person (Russell 2011).

What seems to be a clear advantage of virtue ethics is shown by Armstrong via Hurstbourne (1999) that it seems able to recognise the realities of everyday nursing practice and nurses' experiences when making moral decisions. By replacing deontic concepts at least in a strong sense, if not totally, he rejects the idea he thinks implicit in obligation act‐centred theories that an action if morally permissible must be right and the agent cannot be blamed. Thus, Armstrong claims they cannot or do not give any or sufficient moral account of remainder (mentioned above). And cannot recognise irresolvable tragic dilemmas. He emphasises the morally relevant role of emotions in such cases recognised by virtue ethics. But again, a question arises as to how much relevance should be given to the emotions in practical reasoning or virtue ethics. Aristotle and Nussbaum (2012) acknowledge they can be misleading and need at least ‘infusion’ with reason.

Another way the role of a nurse is important for Armstrong's work occurs when he uses MacIntyre's (1985) idea of a practice to give context to the virtues necessary for nursing. Flourishing (faring well as *eudaimonia*) grounds aside, MacIntyre's technical account of a practice and internal goods requires Armstrong to be able to state the internal goods and *telos* of nursing practice. Although Armstrong criticises MacIntyre's claim that only people within the practice can understand the goods necessary for it and uses instead the notion of purposive practice, practices with specific social purposes, whereby people not part of the practice can judge the goods, the problem that the very idea of a practice was supposed to help resolve, that of a variety of goods and loss of context for virtues must begin to resurface as one of there being many goods and many contexts within nursing (and in Armstrong's case without nursing as well).

Additionally, there may be a clash between social goods and ethical/moral goods (Swanton 2016). Thus, there may be no shared conception of the good life in/for nursing that Armstrong claims and further he claims that what goods there are can and will change over time. What might be seen as ‘bad’ by some within or in Armstrong's case without the practice might be seen as ‘good’, and there may be no way to evaluate each other's internal goods or practice dependent on how strongly contextualist and particularist Armstrong thinks morality or nursing ethics is. We seem to be heading to full blown moral relativism and the loss of morality altogether. The position of MacIntyre on evaluative critique within and between traditions is of course much more complex, but relativism does loom large.

Armstrong (2007, 121) also struggles to state what nursing's internal goods are but gives three: the development and sustenance of a virtue‐based helping relationship, the exercise of moral wisdom and feeling valued by patients and their relatives. None of the three seem specific to nurse's role(s) and the latter seems to make value of nursing internal to nurses themselves rather than for the good of the patients as for example in patient‐centred care that Armstrong emphasises.

Additionally, Armstrong is explicit that as a virtue ethics all evaluation is in terms of the agent's character or virtue. On Swanton's target‐centred approach this is not the case (nor I think is it the case in MacIntyre especially his later work).

The target of a caring doctor in assessing a patient is not to make her feel good but to give an accurate diagnosis, but in a respectful way.

(Swanton 2016, 694)

Here, however, it seems to me at least that it will be just as hard to state nursing's target(s) just as it is to state their internal goods.

A somewhat more indirect point about the need to understand a nurses' role is where Armstrong claims the moral importance of the nurse–patient relationship especially listening to and making sense of the patient narrative about their illness. The interpersonal responses a nurse ought to demonstrate are based upon the claim that the nurse–patient relationship is an unequal one especially in terms of knowledge and power. In fact, Armstrong (2007, 10) citing Pellegrino and Thomasma (1993), claims the inequality is ‘paralleled by few other situations in democratic society’. This may have been true of medicine, but I wonder if it is so today and especially in regard to the knowledge that nurses have. The interpersonal responses claimed necessary for nurses are overall quite general claims that professionals, including healthcare professionals, should respond to patients as individuals showing respect and also demonstrating moral virtues (Armstrong 2007, 19). A further, more contentious, claim is

The above conception of the nurse–patient relationship is held by patients to be extremely valuable, as valuable, if not more valuable, than other clinical interventions.

(Armstrong 2007, 112)

This is a contingent matter, and the study cited in support of this claim was about mental health nurses. Here, I think, the nurse–patient relationship and the necessary virtues are in fact a part of the ‘clinical intervention’.

Armstrong claims nurses ought to demonstrate certain character traits as virtues. However, the character traits that nurses ought to demonstrate are unsurprising and somewhat platitudinous. Armstrong (2006, 112) claims kindness and honesty are important for the development of a therapeutic nurse–patient relationship and kindness, patience and tolerance contribute to high‐quality care, and compassion is essential for nurses to provide morally good care. Presumably, it will not be compassion as Aristotle understands it where the recipient must be deserving of compassion. Later in the paper, he also claims nurses need to exhibit a wide range of moral virtues and avoids trying to list what they are. This when combined with Armstrong's particularism and contextualism makes it very hard to understand how anyone could know either. An answer seems to be the use of *phronesis*. As mentioned, Armstrong emphasises the need for, thinking hard about virtue and vice terms, judgement and *phronesis* though the latter would entail the first two.

*Phronesis* solves Aristotle's mean whereby nurses should demonstrate virtues in the right place and the right time to the right extent, etc. As should everyone else. And this Aristotelian insight is directly related to the idea of decision making. It is one thing to say the *phronimos* could demonstrate this but quite another that most of us ever could. Armstrong (2007) himself denies the usefulness of this for nurses. But he also does not want to limit the virtues for nurses to just a few. But how then are nurses to know what to do?

Swanton (2016, 2021) gives an interesting account of *phronesis* for a non *eudiamonistic* pluralistic virtue ethics that is, I think, relevant to nursing and nurses' roles. On this virtue ethics approach, roles are part of morality (ethics) not abstracted away from being a good human being (Swanton 2016; Annas 2024). But neither need role virtue be grounded in the very general account of what it is to be a good human being. Practical wisdom (*phronesis*) ensures roles are given their appropriate weight in ethics.

Importantly, *Phronesis* on Swanton's account integrates all practical reasons

Virtue ‘proper’ such as generosity proper, is generosity in which all fields of the practical relevant to excellence in an agent's generosity (such as the narrative particularities of her life, her cultural location and her roles) are integrated in an excellent or good enough way.

(Swanton 2016, 702)

So, Swanton's account of ethics (distinct from morality [Williams 1985; Queloz and van Ackeren 2024]) just is (to me at least) practical reason. And *phronesis* is fallible requiring role virtues which is to have expertise in a role.

We do not want virtuous agents of practical wisdom tout court but without relevant expertise bumbling around as alleged ethical experts in the fields of medicine, law and business.

(Swanton 2016, 691)

There is a unity of virtue understood as basic virtue which stays at a very general level and something we all can have with *phronesis*, but ‘we’ cannot all have role virtue as there are just too many and we cannot be experts in all roles. But now we are back to the problem of stating what nursing's expertise is. As Armstrong states, it will be difficult. In fact, it seems so difficult I wonder if its nature exists.

In sum, Armstrong's work on virtue ethics for nursing is important. However, much remains contested such as the importance of the psychological dispositions as character traits for morality or ethics. Whether a virtue ethics can give clearer action guidance and assessment than obligation act‐centred theories is contested. As is a virtue ethics grounded in naturalistic account of *eudaimonia*. Perhaps Swanton's pluralistic, non‐*eudaimonistic*, target‐centred virtue ethics provides a better account. With its clear distinction of morality and ethics, its account of *phronesis* as practical reason and how it makes roles a part of it all is just what Armstrong is after for nursing practice and nursing ethics. In a sense, it seems to have ‘de moralised’ morality. In healthcare, this is no bad thing.

## Conflicts of Interest

The author declares no conflicts of interest.

## Data Availability

The author has nothing to report.
